# Use of simulation scenarios and vote cards in teaching critical appraisal concepts in evidence-based medicine

**DOI:** 10.1186/s12909-023-04738-8

**Published:** 2023-10-04

**Authors:** Ashleigh Peng Lin, Yun-Yun Chou, Ka-Wai Tam

**Affiliations:** 1 Department of Medical Education, Taipei Veterans General Hospital, Taipei, Taiwan; 2 Shared Decision Making Resource Center, Shuang Ho Hospital, Taipei Medical University, New Taipei City, Taiwan; 3 Division of General Surgery, Department of Surgery, Shuang Ho Hospital, Taipei Medical University, New Taipei City, Taiwan; 4 Division of General Surgery, Department of Surgery, School of Medicine, College of Medicine, Taipei Medical University, Taipei, Taiwan; 5 Cochrane Taiwan, Taipei Medical University, Taipei, Taiwan

**Keywords:** Critical appraisal, Evidence-based medicine, Risk of bias, Simulation scenarios, Undergraduate medical education, Vote card

## Abstract

**Background:**

The most effective method of teaching critical appraisal concepts remains unclear. We used simulation scenarios in a Risk-of-Bias (RoB) 2.0 framework to teach the various biases that may affect randomized controlled trials and assessed whether including this interactive session in an evidence-based medicine (EBM) course for third-year preclinical medical students can optimize their understanding of critical appraisal concepts.

**Methods:**

The session had 13 modules, each corresponding to a particular risk of bias in RoB 2.0. Each module included a simulated scenario, followed by data presentation and a generalized conclusion. The students were subsequently asked to use colored vote cards to indicate whether they agreed, had some concern, or disagreed with the conclusion and to justify their answers. On the basis of the students’ answers, the facilitator debriefed the scenario and addressed the specific bias. In each module, the students were required to demonstrate critical thinking in analyzing the claims and quality of the supporting evidence and in justifying their decisions, thus conceptualizing their understanding of research biases.

**Results:**

We included 306 students across two pilot sessions in spring 2020 and 2021, and the response rate was 97.4%. The students were least able to discern the following problems: baseline imbalances when assessing allocation bias (correct answers: 9.06%), missing outcome data when assessing attrition bias (correct answers: 11.65%), and balanced nonprotocol interventions when assessing performance bias (correct answers: 14.88%). The postcourse survey revealed several aspects of the interactive session that the students appreciated or found challenging.

**Conclusion:**

Preclinical medical students generally appreciated the inclusion of simulation scenarios and vote cards in an EBM course. The use of vote cards facilitated medical students’ understanding of critical appraisal concepts, uncovered areas that they found challenging to understand, and encouraged their active participation. Such interactive sessions should be increasingly included in medical education.

**Supplementary Information:**

The online version contains supplementary material available at 10.1186/s12909-023-04738-8.

## Introduction

Critical appraisal is the process of carefully and systematically examining research to judge its trustworthiness, value, and relevance in a particular context [1]. Learning how to independently and appropriately use information is an essential component of medical education and can reduce medical errors, promote individualized care, and increase application of best practices [2–4]. However, the sheer volume of literature, further compounded by the COVID-19 pandemic, can hinder the effective use of information [5–7]. Despite the benefits and importance of developing such skills, medical students struggle with critical appraisal skills [8]. Therefore, there is a need to implement new educational approaches to teach critical appraisal skills in undergraduate medical education (UME) programs.

The optimal delivery of critical appraisal training remains undetermined. Traditionally, didactic lectures and journal clubs have been used to teach critical appraisal skills [9]. However, these can be passive experiences for learners who are not actively involved in the preparation of teaching materials. Additionally, basic critical appraisal concepts can be abstract and challenging for medical students to grasp when taught in the traditional manner, and standalone teaching programs improve student knowledge but not their skills, attitudes, or behavior, especially in medical students with limited exposure to research methodology [10]. More recently, clinically-attached student-led presentations, small group discussions, and team-based learning have been proposed as educational strategies to effectively teach evidence-based medicine (EBM) in UME [11–13]. The use of interactive and clinically integrated learning activities alongside supportive information assists learners in creating a mental model, which may enhance learning and clinical judgement [4]. Critical appraisal content should therefore be delivered using relevant clinical scenarios and interactive modules to foster higher-order thinking.

Randomized controlled trials (RCTs) are the predominant study for teaching critical appraisal skills, as they are the gold standard for evaluating the effectiveness of interventions. Recognizing the various biases in RCTs is essential in medical education; failure to do so can lead to wrong conclusions about the benefits and harms of interventions [14]. Cochrane’s risk-of-bias 2.0 (RoB 2.0) tool organizes the various biases in clinical trials into fixed sets of domains, which focus on different aspects of trial design, conduct, and reporting [15]. Within each domain, users of RoB 2.0 answer one or more signaling questions, which lead to judgments of “low risk,” “some concern,” or “high risk” of bias. Despite this structured approach, medical students can get lost in the convoluted algorithm and fail to recognize the abstract biases arising from a trial.

Simulation scenarios with vote cards may provide the scaffold and support trainees’ need to understand and apply critical appraisal concepts. Using realistic scenarios, the facilitator can translate abstract ideas into concrete concepts. In addition, studying clinically relevant scenarios helps the learner associate the material with activities they will experience in future practice [16]. The concurrent use of vote cards with such scenarios can encourage active participation [17]. Throughout the interactive process, the assessor can also determine which aspects of the scenario may prove most challenging and provide specific feedback to close a knowledge gap [18]. Because of these advantages, simulation scenarios with vote cards may be a means to foster deeper learning of critical appraisal concepts.

At present, the most effective method for teaching critical appraisal concepts remains unclear [19–22]. Although interactive and clinically-relevant sessions show promise, few studies have focused on students’ cognitive understanding of various biases and their perceptions of interactive sessions on learning critical appraisal concepts, especially in preclinical students with limited clinical exposure. In this study, we developed an innovative session, using simulation scenarios and vote cards in a RoB 2.0 framework, to teach the various biases that may affect RCTs and assessed whether including this interactive session in an EBM course for medical students could improve their understanding of critical appraisal concepts. This study aimed to: (a) identify the most challenging biases that preclinical students face; (b) gain a better understanding of preclinical students’ perceptions on the structure, delivery, and impact of this interactive module on their learning of these abstract concepts.

## Methods

### EBM course and learning objectives

The Doctor of Medicine program at xxxxxx University School of Medicine is a six-year undergraduate degree that consists of four preclinical years followed by two years of supervised clinical placements. EBM is a mandatory third-year undergraduate course intended to teach medical students critical appraisal concepts in preparation for future application in healthcare decision-making. Although the course has been well received by students, some have requested teaching that goes beyond recall-based knowledge, captures critical thinking, and provides reinforcing interactions. The need for application, rather than knowledge recall, of critical appraisal concepts has been further exemplified by the COVID-19 pandemic, during which health-care professionals have depended on the rapid assessment of data emerging daily to guide treatment [7]. Accordingly, in 2019, we joined an institution-wide endeavor aimed at improving course content to align with critical thinking outcomes. Designing and implementing simulation scenarios and vote cards to teach critical appraisal concepts was our contribution to this institutional goal. Our local institutional research board approved the project (Approval Number: N202007041), which was delivered in-person in the spring semesters of 2020 and 2021.

We developed an in-class interactive session using simulation scenarios and vote cards to reinforce students’ critical appraisal knowledge and skills while fostering interaction in a supportive learning environment. The objectives of this session were as follows:

1. Learn critical appraisal through bias-embedded scenarios.
2. Analyze the claims based on data presented in the scenarios.
3. Express opinions by using vote cards and justify the response.
4. Conceptualize abstract concepts of biases in RCTs using the RoB 2.0 framework.

## Study design and participants

This study is a one-group post-test only design that sought to measure students’ perception of the effectiveness of an interactive teaching session on their learning of critical appraisal concepts. The sessions were delivered to the entire class simultaneously and in-person across two cohorts of third-year preclinical medical students (2020 and 2021). The team of facilitators consisted of full-time faculty members who were trained in EBM, and who constituted the EBM team at xxx University.

### Innovative teaching session

The course on knowledge of the biases arising from RCTs and the RoB 2.0 tool was delivered as a 2-h didactic lecture 1 week prior to the interactive session. In the following week, a 2-h interactive session was given. This session had 13 modules, each corresponding to a particular risk of bias. Each module was delivered via powerpoint (Figure S1) and started with the facilitator briefly introducing the scenario. The facilitator subsequently presented pertinent data, which primed the students to look for specific strengths and weaknesses germane to the scenario under consideration. The facilitator drew a conclusion based on the data and asked students whether they “agree,” “have some concern,” or “disagree” with the conclusion. The students were given 1 min to ponder the scenario, vote, and justify their answers. Students wrote down their answers on paper and voted accordingly using physical coloured ‘vote cards’ (green/yellow/red), 15 × 11 cm in size, that corresponded to “agreeing”, “having some concern”, or “disagreeing” with the statement presented. The answers of each module were recorded on paper and served as a surrogate of the effectiveness of the didactic lecture. Students were discouraged from cheating as their assessments would remain anonymous and not affect their grades in the course. On the basis of the students’ answers, the facilitator debriefed the scenario and addressed the specific bias. The 3D (defusing, discovering, and deepening) model of debriefing was used to guide the debriefing session [23]. Debriefing allowed the students to consider their strengths and areas for improvement. Linking biases to scenarios fostered a concrete understanding of biases and prevented students from being confused by a list of biases that can be easily forgotten. At the end of each module, the students were asked whether their understanding of bias would change their decisions. Throughout each module, the students were required to demonstrate critical thinking when analyzing the claims and quality of the supporting evidence and when justifying their decisions, both of which helped them to conceptualize their understanding of research biases.

### Simulation scenarios and vote cards

The design and development of the simulation scenario and the vote card system were based on the RoB 2.0 tool, which is used to assess the risk of bias in RCTs across five distinct domains: (a) bias arising from the randomization process, (b) bias due to deviations from intended interventions, (c) bias due to missing outcome data, (d) bias in measuring the outcome, and (e) bias in selecting the reported result. Within each domain, users answer one or more signaling questions that aim to elicit information relevant to the assessment of risk of bias. The answers lead to judgments of “low risk of bias,” “some concerns,” or “high risk of bias.” The judgments within each domain lead to an overall risk-of-bias judgment of the result being assessed. The 13 scenarios were developed parallel to this framework, each of which corresponded to an important risk of bias (details in Figure S1). Seven course instructors provided independent subjective ratings of the scenarios and reconciled their impressions through consensus. For each scenario, the students used different-colored vote cards to express agreement, having some concern, or disagreement with the conclusions presented. For example, to demonstrate missing outcome data when assessing attrition bias, we asked students to imagine being an endocrinologist investigating the efficacy of a new antidiabetic medication in reducing blood glucose levels. One week into the trial, seven patients in the new medication group developed a severe allergy and received the old medication for the remainder of the trial. The students were asked whether the data for those seven patients should be managed in the new or old medication group or censored. On the basis of the vote card results, the facilitator gave a short debriefing session.

### Student feedback on interactive session

Following session conclusion, all students completed a paper evaluation form about their experiences with the simulation scenario and vote card system. The survey questions were developed on the basis of the course learning objectives and were rated on a 5-point Likert scale (1 = *strongly disagree*, 5 = *strongly agree*). In addition, we asked students open-ended questions about their challenges and successes during the modules.

### Statistical analysis

The percentage of correct answers corresponding to each module was calculated by dividing the number of correct answers by total responses. The postcourse survey statements reflecting students’ perception of the course were categorized into reaction, attitude, and confidence. The mean evaluation scores and standard deviation of each statement was calculated. Missing data for each question was not included. Cronbach’s alpha was calculated to evaluate the reliability of the survey. In this study, Cronbach’s alpha of the survey was 0.912, indicating a high level of internal consistency. Data were analyzed using SPSS version 18.0 software (SPSS Inc, Chicago, IL).

## Results

We enrolled 306 third-year medical students across two sessions (153 students in 2020, 153 students in 2021), with 298 (97.4%; 63.1% women) completing both the course evaluation and survey, and 62 (20.3%; 25 students in 2020, 37 students in 2021) providing open-ended feedback.

The analysis of the students’ vote card responses revealed that the students were least able to discern the following problems: baseline imbalances when assessing allocation bias (correct answers: 9.06%; Table 1), missing outcome data when assessing attrition bias (correct answers: 11.65%), and balanced nonprotocol interventions when assessing performance bias (correct answers: 14.88%). By contrast, the students were most able to identify the following biases: participant or personnel awareness of interventions when assessing performance bias (correct answers: 95.79%), outcome assessor awareness of intervention when assessing measurement bias (correct answers: 85.11%), and randomization of sequence when assessing allocation bias (correct answers: 81.23%).

**Table 1:** Percentages of medical students (n = 306) who correctly answered questions on different scenarios testing their knowledge of the five domains of biases listed in Risk-of-Bias 2.0

Scenario topic*	Medical students (n = 306)	
	% of correct answers	
Allocation bias Randomization of sequence	81.23	
Concealment of allocation	61.49	
Problems of baseline imbalances	9.06	
**Performance bias** Participant/personnel awareness of intervention	95.79	
Balanced nonprotocol interventions	14.88	
Deviation from intended interventions	55.67	
**Attrition bias** Missing outcome data	11.65	
Evidence that result is not biased	57.23	
Missing data could depend on true value	16.45	
**Measurement bias** Inappropriate measurement of outcome Outcome assessor awareness of intervention	26.54 85.11	
**Reporting bias**		
Results selected from multiple outcome measurements	76.38	
Results selected from multiple analyses of the data	53.37	

*Derived from the five domains of biases listed in Risk-of-Bias 2.0

Postcourse surveys were designed to measure student reactions, attitudes, and confidence in recognizing the various biases (Table 2). The results revealed that most students enjoyed the use of simulated scenarios and vote cards in learning critical appraisal concepts (mean: 4.49, standard deviation [SD]: 0.75), that using real-world examples allowed them to grasp the importance of recognizing biases (mean: 4.34, SD: 0.73), and that they were more confident in their ability to recognize the various biases (mean: 3.89, SD: 0.79). Open-ended feedback was overwhelmingly positive (Table 3). The students revealed that they enjoyed applying critical appraisal knowledge to simulated scenarios, that debriefing sessions from the facilitator addressed gaps in knowledge, and that interactive sessions encouraged active participation and deeper learning.

**Table 2:** Use of simulated scenarios and vote cards: Reaction, attitude, and confidence in bias recognition of medical students

	Item	Mean	SD
Reaction	The simulated scenarios clearly reflected each domain of bias	4.31	0.74
	The use of vote cards allowed me to pay attention to important concepts	4.45	0.75
	Vote cards effectively promoted engagement in interactive dialogue with both the facilitator and peers	4.44	0.71
	The use of vote cards increased my ability to focus on the critical thinking process	4.41	0.74
	Vote cards facilitated useful immediate feedback and live interactions	4.57	0.66
	Compared with hand-raising, the use of vote cards was better at reinforcing critical appraisal concepts	4.57	0.66
	Compared with didactic lectures, using simulated scenarios and vote cards is more beneficial to my learning	4.49	0.77
	I would recommend the use of simulated scenarios and vote cards to my peers	4.46	0.78
	My program should continue to use this curriculum to teach critical appraisal concepts	4.49	0.75
Attitude	The simulation format facilitated my understanding of the domains of bias in RoB 2.0 and will help me critically appraise future RCT literature	4.29	0.76
	Learning RoB 2.0 through simulated scenarios has helped me learn the importance of recognizing biases in research studies	4.34	0.73
	Compared with didactic lectures, simulated scenarios have increased my understanding of the thinking process behind the design of a research project	4.51	0.68
	Simulated scenarios increased the reality and excitement of understanding critical appraisal concepts	4.62	0.63
Confidence	Identify allocation bias	3.89	0.79
	Identify performance bias	3.87	0.76
	Identify attrition bias	3.73	0.79
	Identify detection bias	3.80	0.79
	Identify reporting bias	3.86	0.78

The items were rated on a 5-point Likert scale (1 = *strongly disagree*, 5 = *strongly agree*)

SD, standard deviation

**Table 3:** Open-ended feedback regarding the interactive session using simulated scenarios and vote cards

Type of comment	
Positive comments	I have gained increased understanding of the importance of critical thinking and what the approach to medical research is and how it can be implemented in the daily work of a physician. I believe that the use of simulation scenarios and vote cards can be the basis for developing critical thinking skills. Through this session, I was able identify my lack of understanding of certain biases. Overall, this interactive session increased my depth of understanding of the various biases in RoB 2.0. This session was engaging and exciting!
Constructive comments	I believe that delivering an innovative interactive online learning platform would allow for more engaging interactions between peers.
	An online format would allow for more flexibility.

## Discussion

Learning theoretical concepts can be challenging for preclinical medical students because of their limited clinical skills and medical knowledge. In this study, we designed an interactive session based on a structured risk-of-bias framework to help third-year preclinical medical students understand critical appraisal concepts. We sought to identify the most challenging biases that preclinical students encounter and gain a better understanding of their perceptions on the structure, delivery, and impact of this interactive module on their learning of these abstract concepts.

In short, the students were able to apply medical knowledge and found the concepts of baseline imbalances, missing outcome data, and balanced nonprotocol interventions most difficult to comprehend. Their inability to recognize these biases after the didactic lecture alone was reflective of theoretical confusion about them, reinforcing previous studies documenting the inherent difficulties in teaching critical appraisal concepts through standalone lectures [22, 24]. We addressed these challenges by using simulated scenarios and vote cards. To our knowledge, this is the first such reported program to use simulated scenarios and vote cards alongside an RoB 2.0 scaffold to translate these abstract concepts into concrete understanding.

Our results revealed that the students frequently failed to recognize the problem of baseline imbalances despite being excellent at discerning sequence randomization and allocation concealment. This may be because the students equated randomization to adequate allocation, leading to a failure to recognize incomparable data in the presence of baseline imbalances. Recognizing baseline imbalances is important because it reduces uncertainty in systematic review conclusions, minimizes the risk of chance findings being ascribed to treatment effects, and contributes to better use of available evidence [25]. Using the RoB 2.0 framework in simulated scenarios allowed students to recognize and comprehend the importance of baseline imbalances.

Regarding attrition bias, the students were often perplexed regarding the management of data when study participants did not adhere to the intended protocol. Beginners often use a per-protocol approach, believing that the patients who violate the research protocol should be excluded from analysis. Compared with an intention-to-treat analysis, a per-protocol analysis can lead to a significantly biased assessment of intervention effectiveness [26]. Learning to analyze results according to the group to which they were originally assigned to is therefore of the utmost importance. In the debriefing session, the facilitator addressed this confusion by explaining the difference between the intention-to-treat principle and per-protocol analysis by using the given simulated scenario. This enabled the students to better comprehend missing outcome data.

In evaluating performance biases, the medical students were aware of the concept of blinding but were confused as to whether bias was introduced when blinding did not affect the outcome measured. Blinding is not always possible and can be heavily dependent on different styles of patient management, surgical procedures, or alternative therapies [27]. In these contexts, conclusions can still be drawn if the lack of blinding does not affect outcomes. Medical students are so accustomed to the concept of double- and triple-blinded clinical trials that they can unintentionally disregard the results of unblinded experiments. Often, however, some limitations of the real world render completely blinded experiments impossible. Through this module, the students were able to appreciate the value of unblinded trials in such conditions.

Overall, the students were satisfied with the interactive session. They felt that the interactive and debriefing sessions improved and deepened their understanding of critical appraisal concepts. We applied the 3D model of debriefing—defusing, discovering, and deepening—all of which enhanced learning after each simulated scenario. During the debriefing period, the students reflected on their reactions to the simulated scenarios, identified the mental models that led to cognitive processes, and described how the particular scenario enhanced their understanding of critical appraisal skills that could be used in future situations. The use of vote cards encouraged students to actively participate in the modules, which we believe was associated with greater satisfaction with and learning of these concepts.

This project had some limitations. First, the medical students were recruited from only one undergraduate program, precluding generalization of the results to other programs or geographic areas. Second, self-reported measures were used for data collection. Third, qualitative data, such as open-ended feedback, was intended to provide an overview of medical students’ perception towards the interactive module. It would be more appropriate if we followed a method, such as thematic analysis, to analyze these qualitative data. Fourth, vote cards were used both for teaching and performance assessment, making it difficult to assess the efficacy of the interactive session. Finally, the project was not designed for statistical significance. Future studies may wish to include additional scenarios with varying levels of difficulty and alternative risk-of-bias frameworks for non-RCTs. Furthermore, this project should be delivered in more medical schools and could even be offered in residency programs and EBM journal clubs to evaluate medical residents’ competency in assessing biases.

## Conclusions

In this study, preclinical medical students generally appreciated the inclusion of simulation scenarios and vote cards in an EBM course. The use of vote cards facilitated medical students’ understanding of critical appraisal concepts, uncovered areas that they found challenging to understand, and encouraged their active participation. A thorough understanding of these concepts will enable the students to avoid these biases when conducting or interpreting research.

### Electronic supplementary material

Below is the link to the electronic supplementary material.

Supplementary Material 1

## Data Availability

The analyzed data used during the study are available from the corresponding author on reasonable request.

## Abbreviations

EBM: Evidence-based medicine
RCTs: Randomized controlled trials
RoB 2.0: Risk-of-bias 2.0
UME: Undergraduate medical education

## References

[1] Burls A (2009). What is critical Appraisal? Evidence-based medicine.

[2] Dawes M, Summerskill W, Glasziou P, Cartabellotta A, Martin J, Hopayian K et al. Second International Conference of Evidence-Based Health Care Teachers and Developers. Sicily statement on evidence-based practice. BMC Med Educ. 2005;5(1):1. 10.1186/1472-6920-5-1 PMC544887 15634359

[3] Albarqouni L, Hoffmann T, Glasziou P (2018). Evidence-based practice educational intervention studies: a systematic review of what is taught and how it is measured. BMC Med Educ.

[4] Maggio LA, ten Cate O, Chen HC, Irby DM, O’Brien BC (2016). Challenges to learning evidence-based medicine and educational approaches to meet these challenges: a qualitative study of selected EBM curricula in U.S. and canadian medical schools. Acad Med.

[5] Druss BG, Marcus SC (2005). Growth and decentralization of the medical literature: implications for evidence-based medicine. J Med Libr Assoc.

[6] Gabriel SE, Normand SL (2012). Getting the methods right — the foundation of patient-centered outcomes research. N Engl J Med.

[7] Cinelli M, Quattrociocchi W, Galeazzi A, Valensise CM, Brugnoli E, Schmidt AL (2020). The COVID-19 social media infodemic. Sci Rep.

[8] Christopher E, Leow HW (2020). Training in critical appraisal skills. Lancet.

[9] Radack KL, Valanis B (1986). Teaching critical appraisal and application of medical literature to clinical problem-solving. J Med Educ.

[10] Coomarasamy A, Khan KS (2004). What is the evidence that postgraduate teaching in evidence based medicine changes anything? A systematic review. BMJ.

[11] Coşkun Ö, Kıyak YS, Budakoğlu Iİ, Ergün MA, Haznedaroğlu Ş (2022). A Novel Approach to teach evidence-based medicine: modified PEARLS. Gazi Med J.

[12] Daou D, Chakhtoura M, El-Yazbi A, Mukherji D, Sbaity E, Refaat MM (2022). Teaching critical appraisal to large classes of undergraduate medical students using team-based learning versus group discussions: a randomized controlled trial. BMC Med Educ.

[13] Stockler MR, March L, Lindley RI, Mellis C (2009). Students’ PEARLS: successfully incorporating evidence-based medicine in medical students’ clinical attachments. Evid Based Med.

[14] Gluud LL (2006). Bias in clinical intervention research. Am J Epidemiol.

[15] Sterne JAC, Savović J, Page MJ, Elbers RG, Blencowe NS, Boutron I (2019). RoB 2: a revised tool for assessing risk of bias in randomised trials. BMJ.

[16] Cook TC, Camp-Spivey LJ (2022). Innovative teaching strategies using simulation for pediatric nursing clinical education during the pandemic: a case study. Acad Med.

[17] Tam KW, Tsai LW, Wu CC, Wei PL, Wei CF, Chen SC (2011). Using vote cards to encourage active participation and to improve critical appraisal skills in evidence-based medicine journal clubs. J Eval Clin Pract.

[18] Rock LK (2021). Communication as a high-stakes clinical skill: just-in-Time simulation and vicarious observational learning to promote patient- and family-centered care and to improve trainee skill. Acad Med.

[19] Horsley T, Hyde C, Santesso N, Parkes J, Milne R, Stewart R (2011). Teaching critical appraisal skills in healthcare settings. Cochrane Database Syst Rev.

[20] Howard B, Diug B, Ilic D (2022). Methods of teaching evidence-based practice: a systematic review. BMC Med Educ.

[21] Ilic D, Maloney S (2014). Methods of teaching medical trainees evidence-based medicine: a systematic review. Med Educ.

[22] Maggio LA, Tannery NH, Chen HC, ten Cate O, O’Brien B (2013). Evidence-based medicine training in undergraduate medical education: a review and critique of the literature published 2006–2011. Acad Med.

[23] Zigmont JJ, Kappus LJ, Sudikoff SN (2011). The 3D model of debriefing: defusing, discovering, and deepening. Semin Perinatol.

[24] Prober CG, Heath C (2012). Lecture halls without lectures–a proposal for medical education. N Engl J Med.

[25] Corbett MS, Higgins JP, Woolacott NF (2014). Assessing baseline imbalance in randomised trials: implications for the Cochrane risk of bias tool. Res Synth Methods.

[26] Abraha I, Cherubini A, Cozzolino F, De Florio R, Luchetta ML, Rimland JM (2015). Deviation from intention to treat analysis in randomised trials and treatment effect estimates: meta-epidemiological study. BMJ.

[27] Day SJ, Altman DG (2000). Statistics notes: blinding in clinical trials and other studies. BMJ.

