# Supplementary material for: Use of simulation scenarios and vote cards in teaching critical appraisal concepts in evidence-based medicine
Source: BMC Med Educ. 2023 Oct 4;23:726. doi: 10.1186/s12909-023-04738-8 (PMC10548740; doi:10.1186/s12909-023-04738-8)

Scenario 1

- You are the principal investigator of a research study comparing two anti-hypertensive medications, one old, the other new. You have one hundred participants. How would you allocate the participants into the two groups?

Group A (old drug)

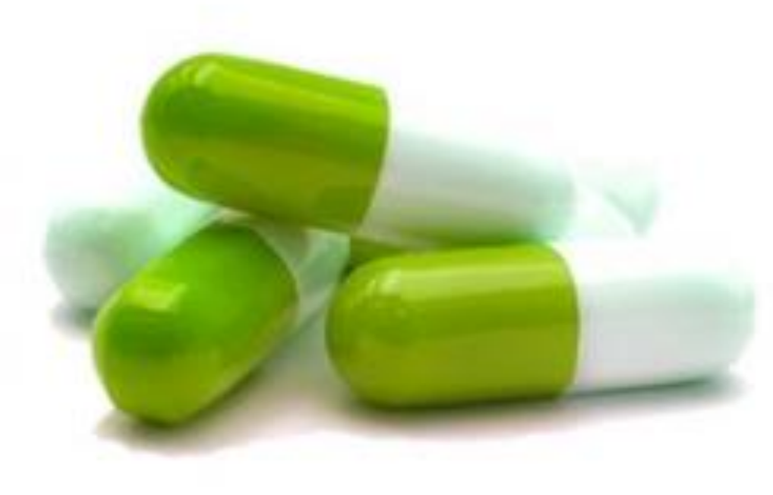

Group B (new drug)

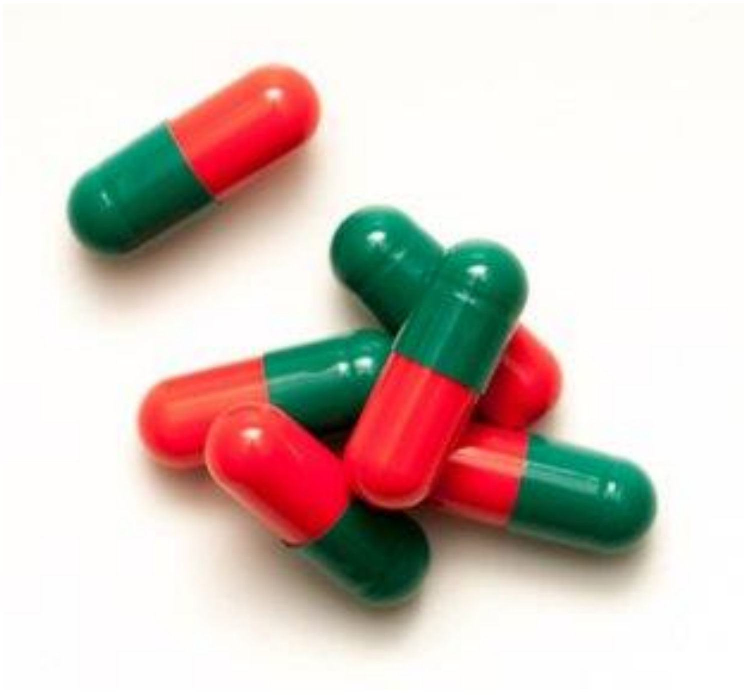

Vote

Physician decision

Computer generation

Patient ID

Scenario 2

- You are the principal investigator of a study investigating the effects of chlorhexidine application before wound closure on wound infection rates. Participants have already been randomly allocated into the experimental and control groups. When would be the appropriate time for you to know the allocation groups of the participants?

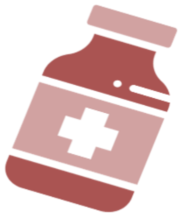

Chlorhexidine

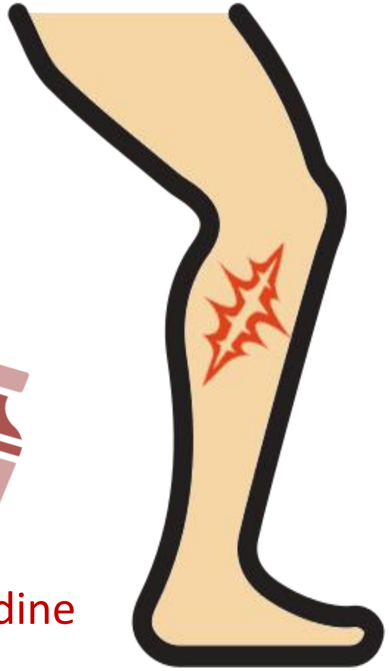

During the patients'…

Outpatient visit

Inpatient visit

Other

Scenario 3

- You are a cardiologist who is planning to perform a percutaneous transluminal coronary angiography (PTCA) and want to know the optimal dressing after the procedure. A recent study compared the efficacy of OpSite and pressure dressing in reducing the risk of hematoma and skin damage.

OpSite Dressing vs Pressure Dressing After Percutaneous Transluminal Coronary Angiography

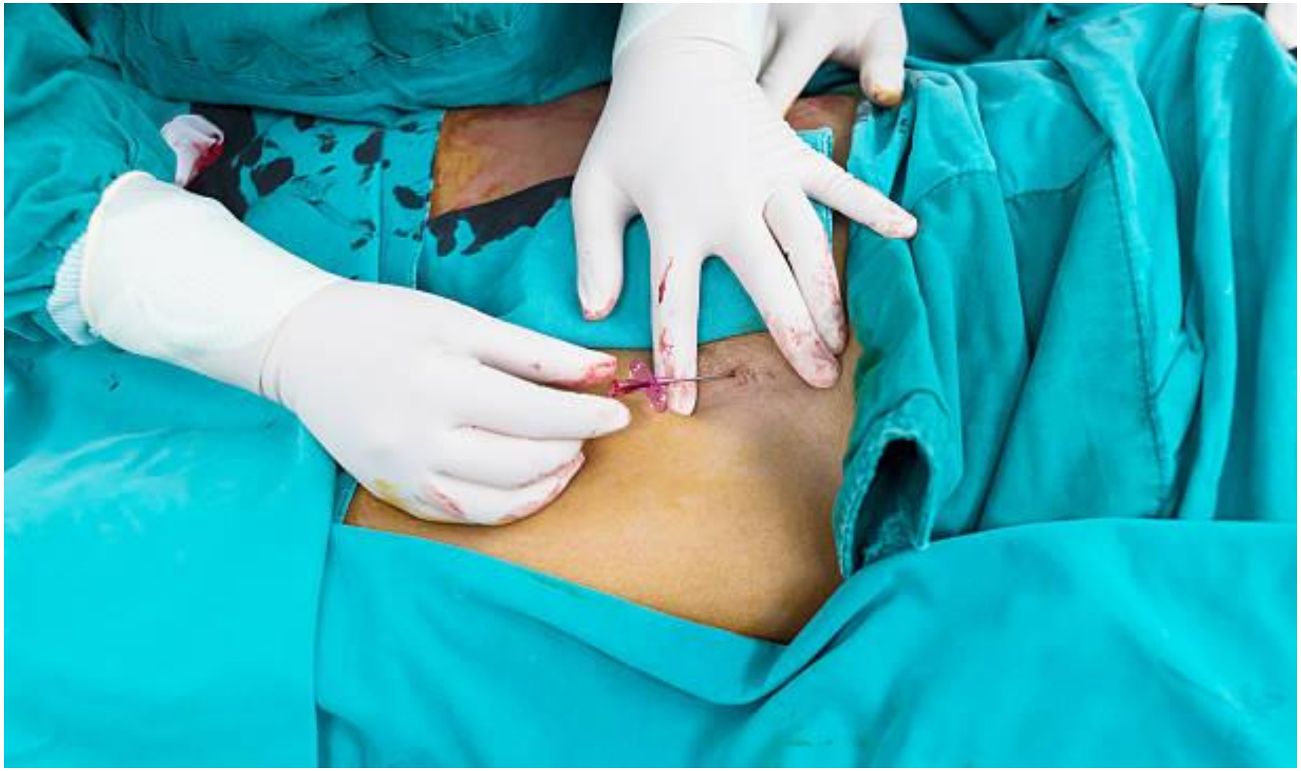

Post-operative dressing

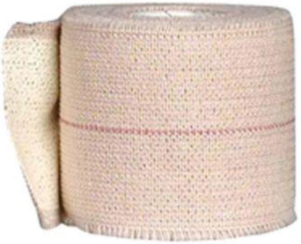

– Pressure dressing group:
  4-in Elastikon (Johnson & Johnson) elastic tape secured over 1 box of 4 x 4-in gauze sponges

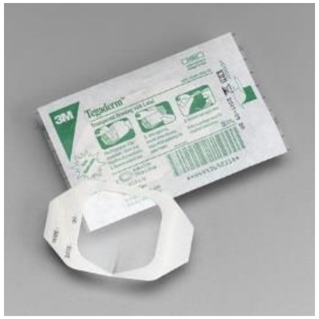

– OpSite group:
  OpSite 4 x 5 -in (10 x 14-cm) 2-in gauze sponges

Demographic data of the sample (N = 100)

Characteristic	Value
Age, mean (SD), y	61 (13.2)
No. of previous catheterizations, mean (SD)	2.05 (2.82)
Activated clotting time, mean (SD), s	164 (20.7)
Sex, % of patients	
Male	67
Female	33
First catheterization, % of patients	
Yes	31
No	66
Drug during angioplasty, % of patients	
Bivalirudin	50
Eptifibatide	32
Abciximab	11
Aspirin, % of patients	
Yes	78
No	18
Clopidogrel, % of patients	
Yes	74
No	25
Type of pressure applied, % of patients	
Manual	61
FemStop device	39

Conclusion

- The study found no significant difference in hematoma rates between the two dressings. However, compared to the use of pressure dressing, the use of OpSite dressing had significantly less skin damage. The study concluded that OpSite should be used as the optimal dressing after PTCA.

Would you agree?

Agree	Doubt	Reject

Figure S1.1 Algorithm for suggested judgement of risk of bias arising from the randomization process.

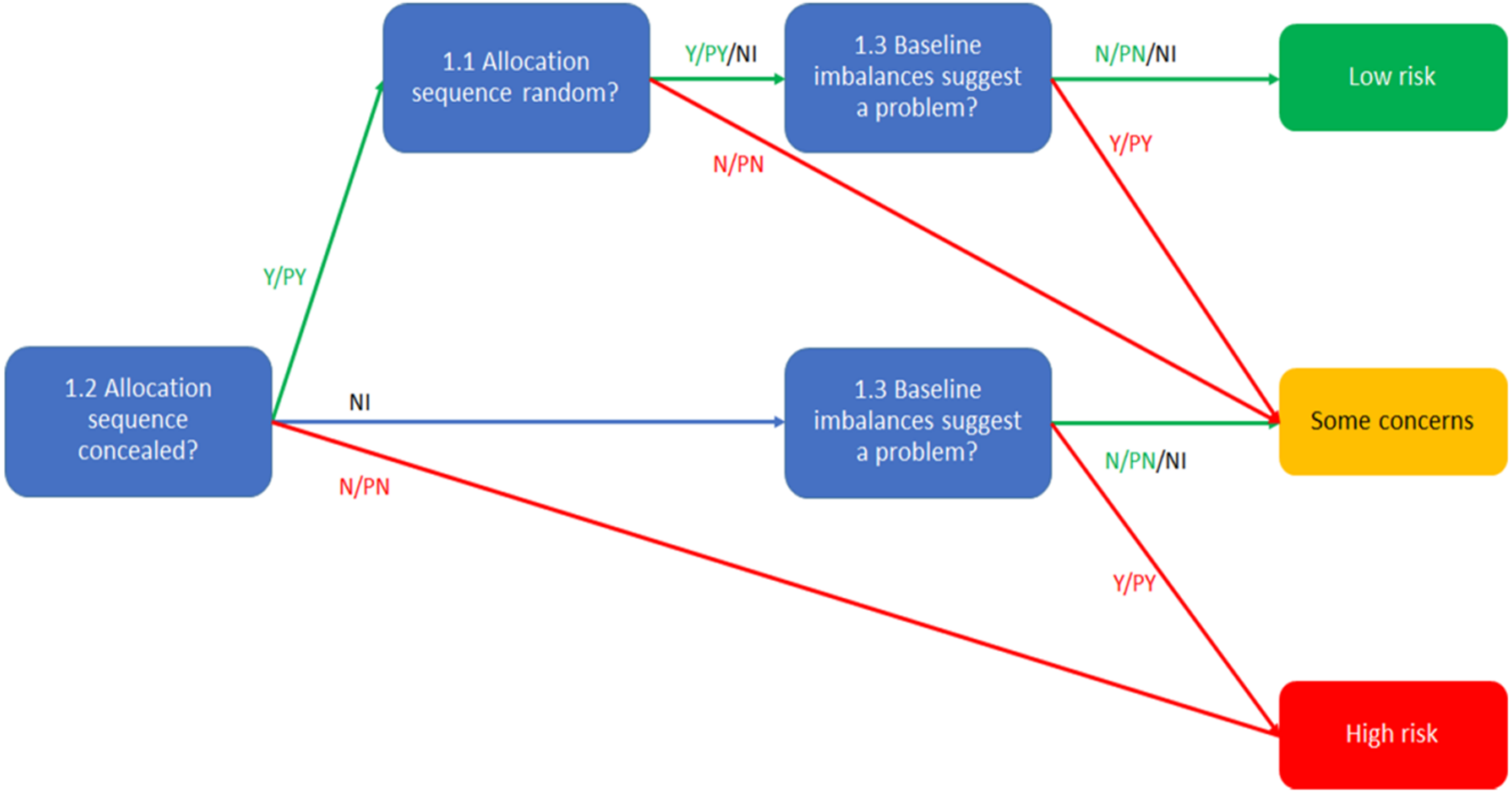

Scenario 4

- You are a gastrointestinologist. A pharmaceutical company has just developed a new prescription weight loss medication and has asked you to investigate its efficacy. You have 100 patients and have randomly assigned 50 patients to the experimental group, what would you use as the control group?

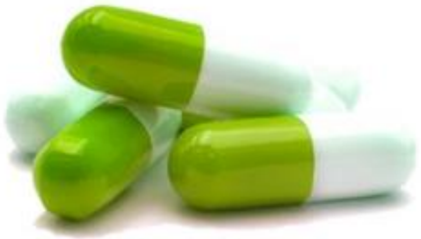

Vote

Nothing

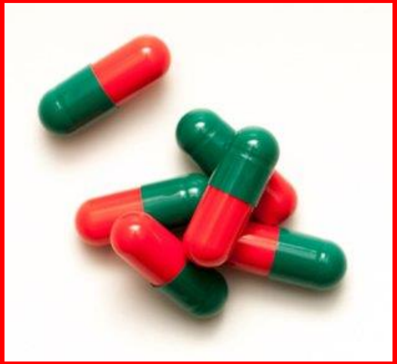

Placebo

Scenario 5

- You are the principal investigator seeking to investigate the effects of listening to classical music for 20 minutes after meal on reducing serum glucose levels in patients with diabetes mellitus. One hundred participants have been randomly allocated into two groups. Fifty participants were assigned to listen to Tchaikovsky’s Swan lake suite. What treatment would the remaining 50 participants receive?

- You have limited financial aid for this study. One hundred participants are all in the same room for the duration of the study.

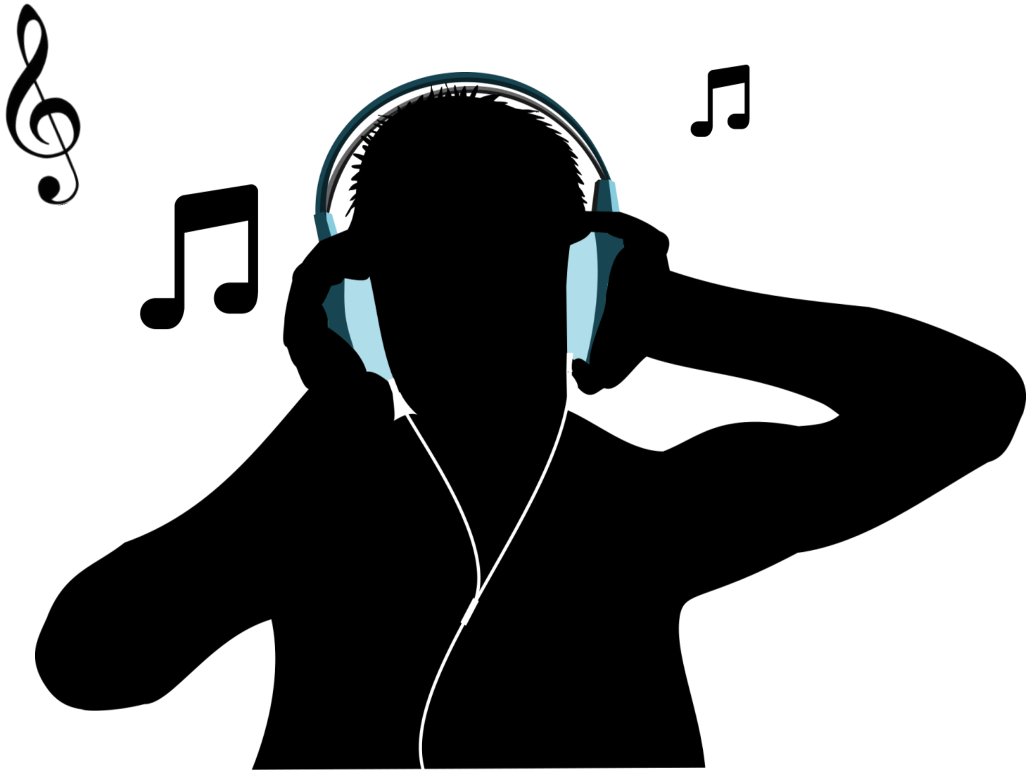

Vote

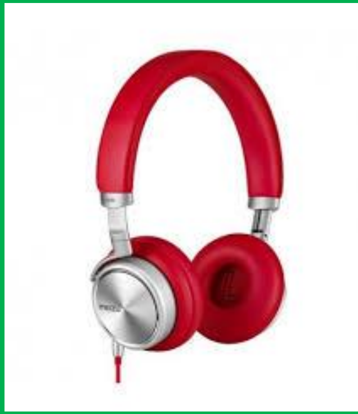

Pop music

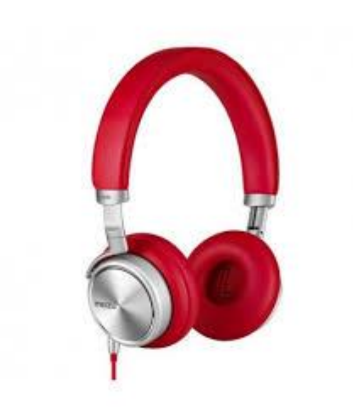

No music

No music and no headphones

Scenario 6

- You are the principle investigator of a study investigating the efficacy of a new experimental spray for reducing post-hemorrhoidectomy pain. You want to measure the 24-hour pain score of the participants. The conditions are as follows:

Spray once right after hemorrhoidectomy

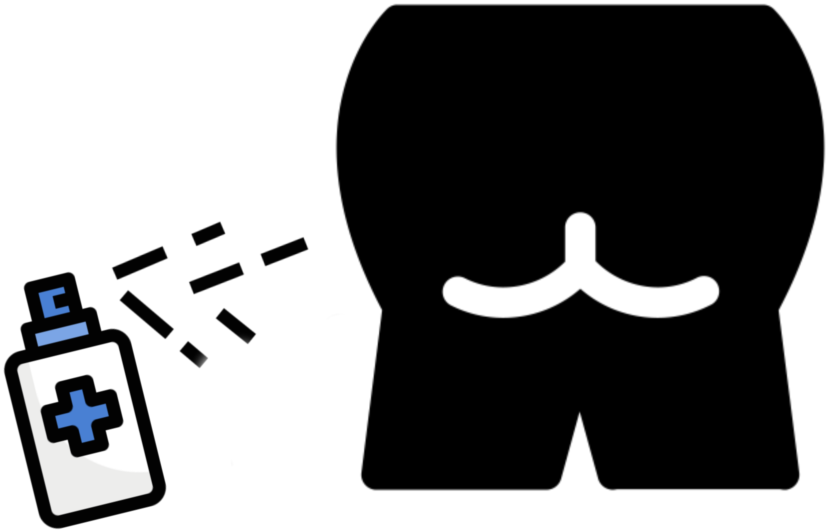

Method :

- Dietary intake 6 hours post-operation
- Single dose of paracetamol Q4H
- If postoperative pain is not relieved, the on-call resident can administer additional analgesics as needed

Would you agree with the above method?

Agree

Doubt

Reject

Figure S1.2 **Algorithm for suggested judgement of risk of bias due to deviations from the intended interventions (*effect adhering to intervention*).** This is only a suggested decision tree: all default judgements can be overridden by assessors.

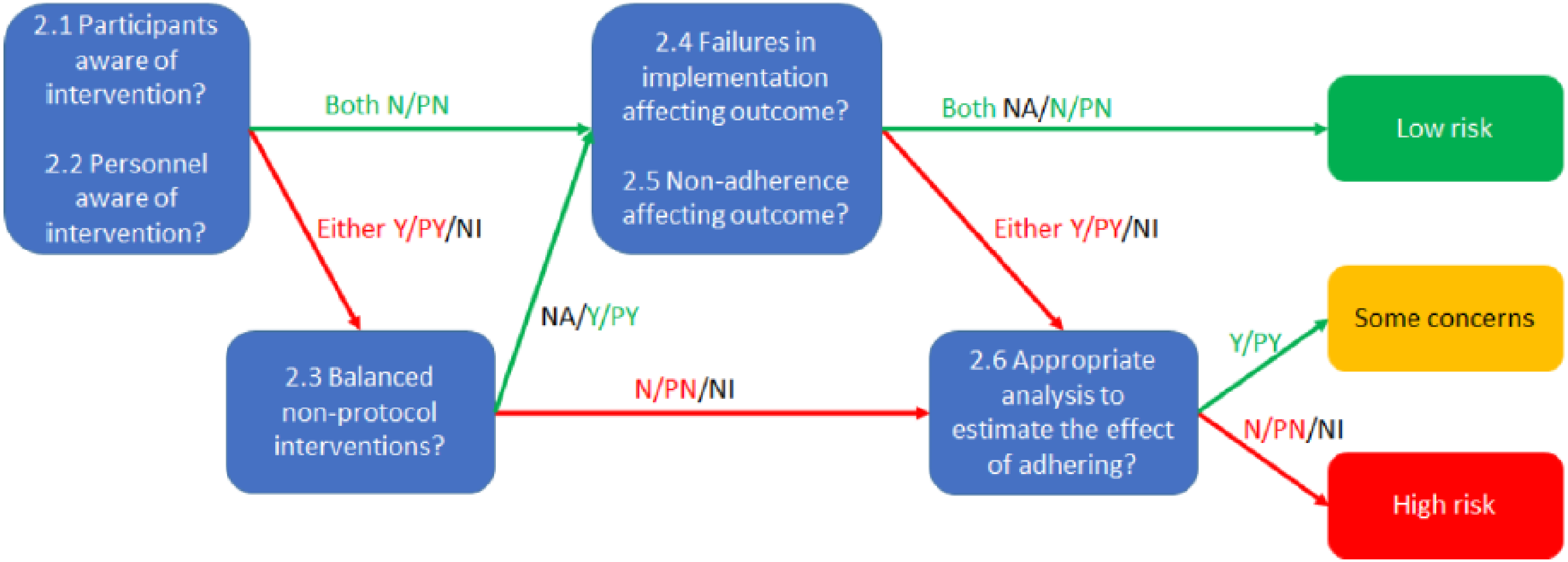

Scenario 7

- You are an endocrinologist evaluating the efficacy of a new anti-diabetic medication in reducing blood glucose levels. There are 200 participants, whom have been randomly allocated into the groups, group A and B (newer). The main outcome is the blood sugar level one month after intervention.

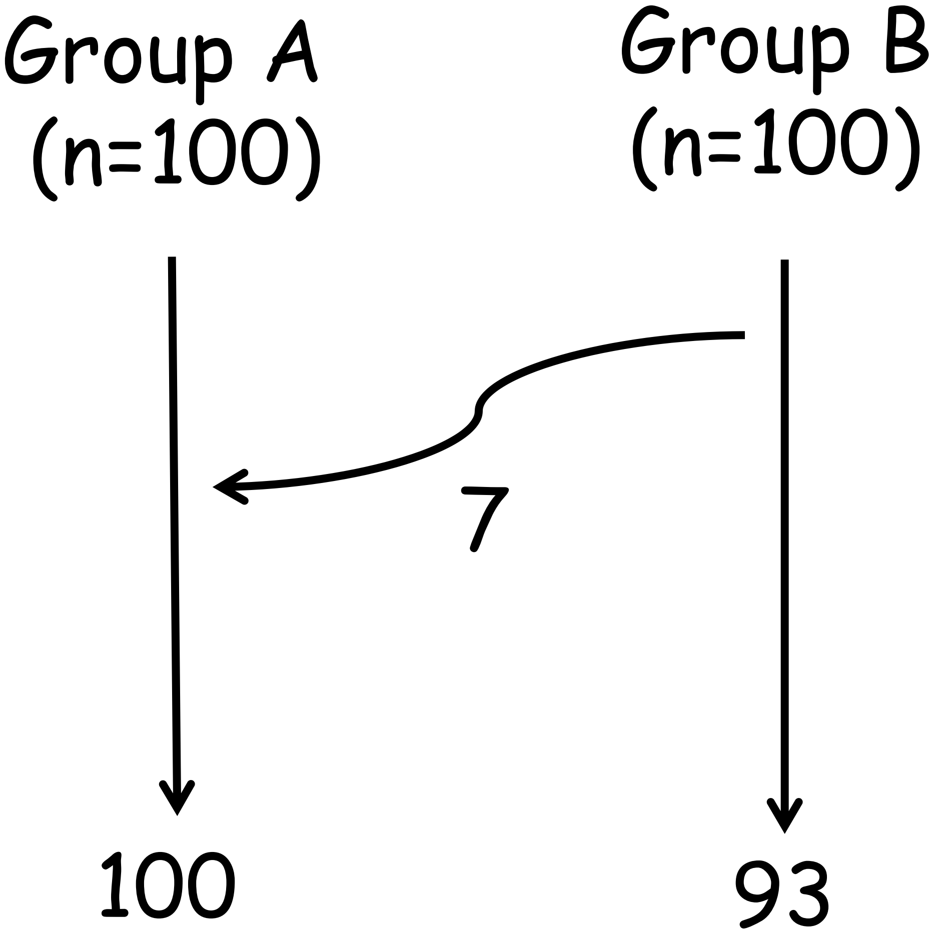

One week into the trial, 7 patients in group B suffered from a severe allergy. For the remaining three weeks, they received the old drug.

How would you manage the blood glucose values of the 7 allergic patients?

B	Drop out	A

Scenario 8

- You are an anesthesiologist evaluating the efficacy of a dose of dexamethasone on reducing postoperative nausea and vomiting (PONV) in patients undergoing thyroidectomy. There are 200 participants, all of whom have been randomly allocated into either the control or dexamethasone group.

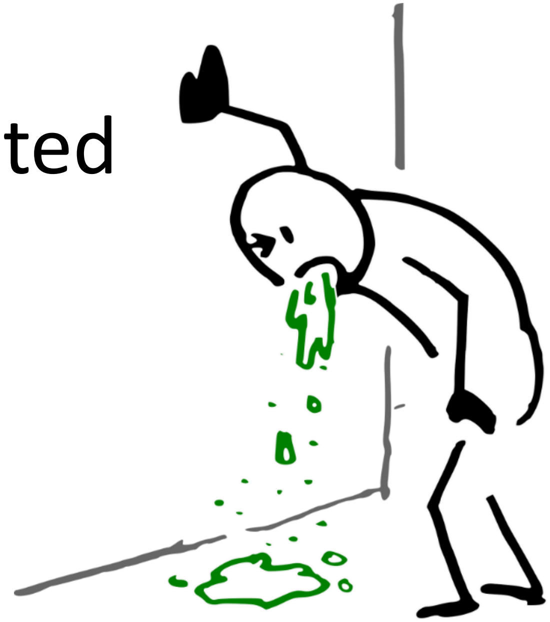

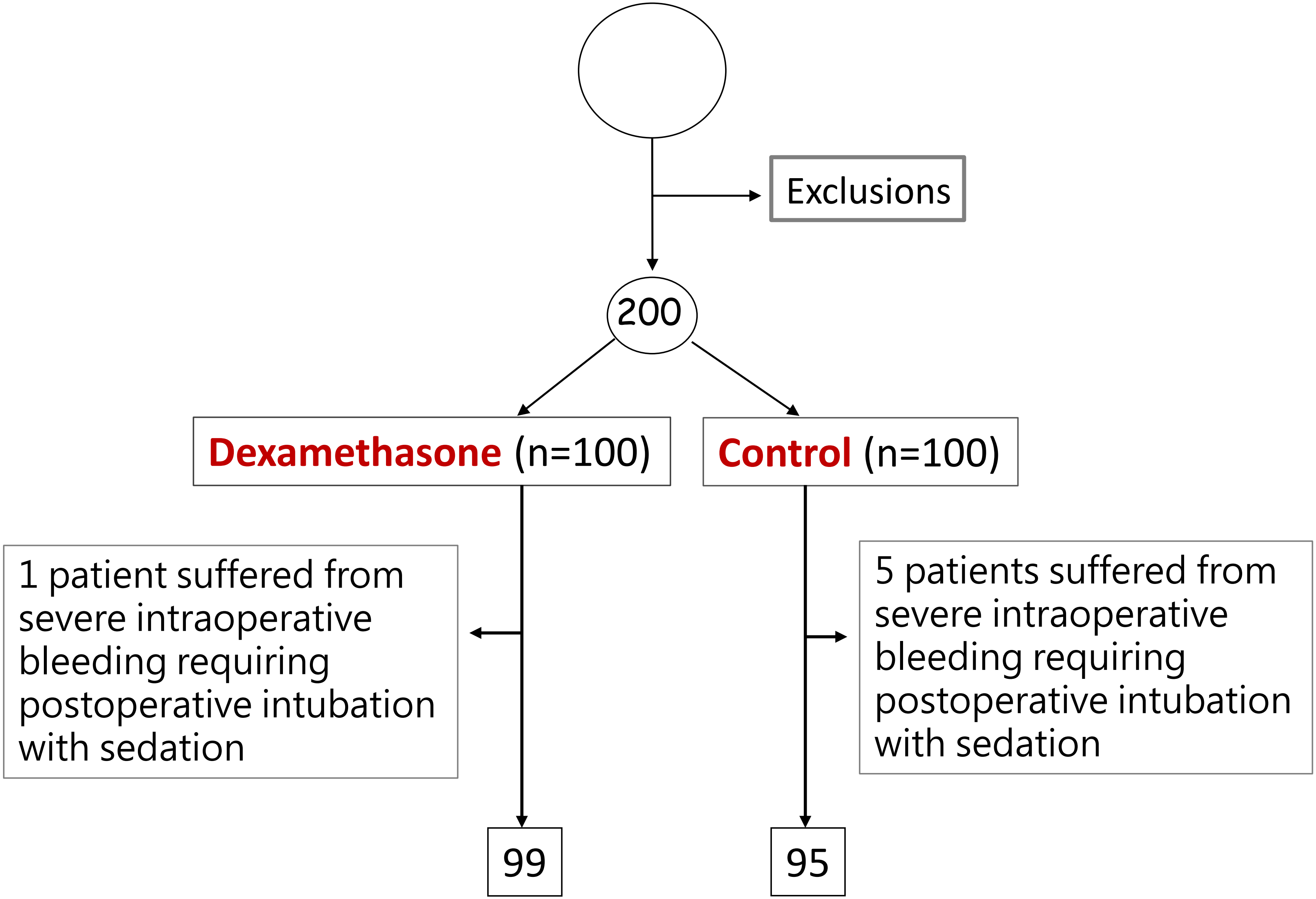
Exclusions
200
Dexamethasone (n=100)
Control (n=100)
1 patient suffered from severe intraoperative bleeding requiring postoperative intubation with sedation
5 patients suffered from severe intraoperative bleeding requiring postoperative intubation with sedation
99
95

Incidence of PONV

Steroid (7/99) vs Control (20/95); P=0.007

Would you agree with the conclusion that the use of steroids significantly reduce the incidence of PONV?

Agree

Doubt

Reject

Incidence of complication

Steroid(n=99) vs Control(n=95)
Each group with n=2 hemorrhage and n=1 infection

Would you agree with the conclusion that steroids are as safe as controls?

Agree

Doubt

Reject

Figure S1.3 . **Algorithm for suggested judgement of risk of bias for bias due to missing outcome data.** This is only a suggested decision tree: all default judgements can be overridden by assessors.

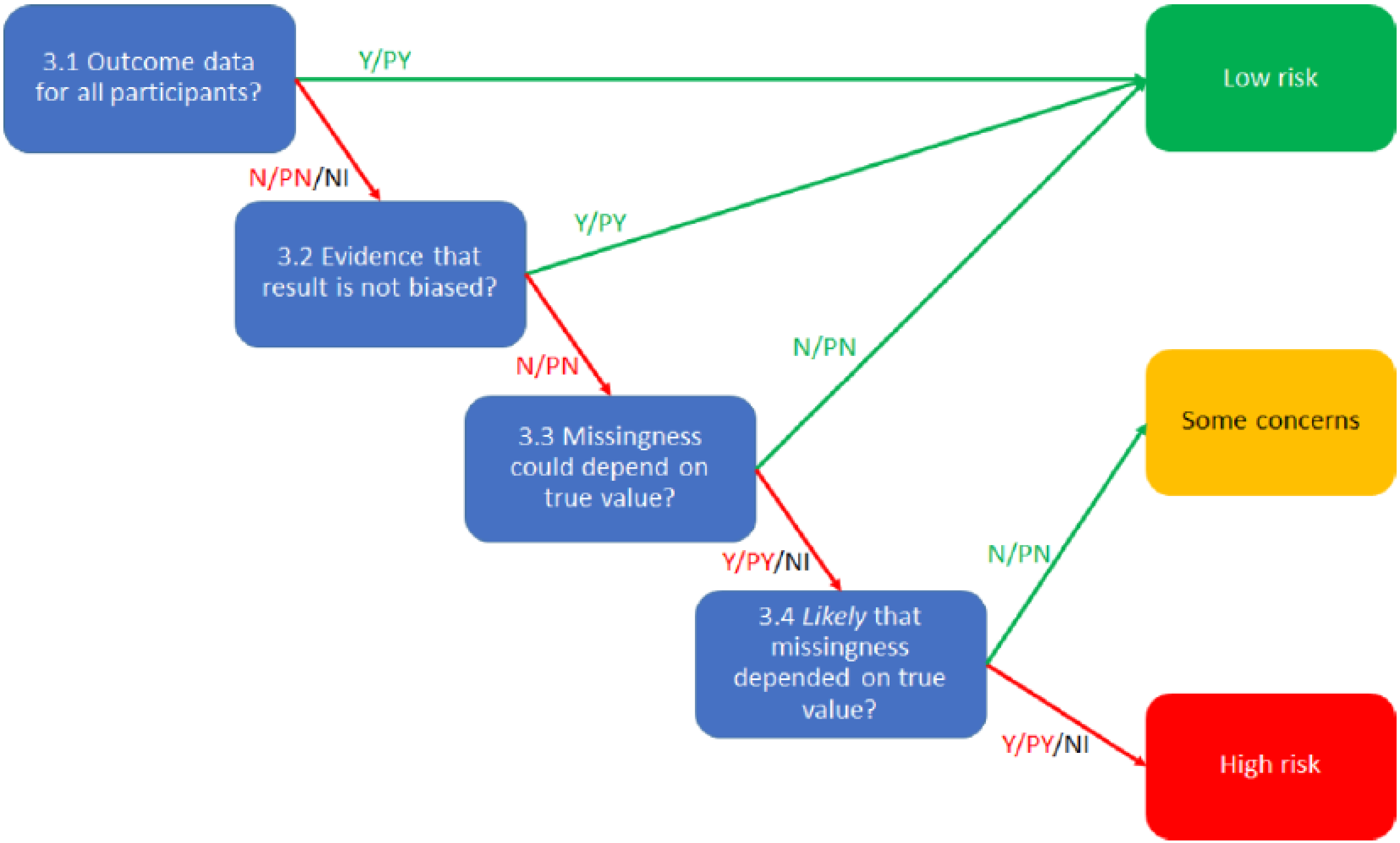

Scenario 10

- There is global pandemic. No known medication has been developed so far to manage this mysterious illness. You are a principle investigator of a randomized clinical trial evaluating the efficacy of a known drug, drug A, vs standard care on the clinical status at day 14 in patients with moderate severity of this condition.

Effect of drug A vs standard care on clinical status at day 15 in patients with moderate disease

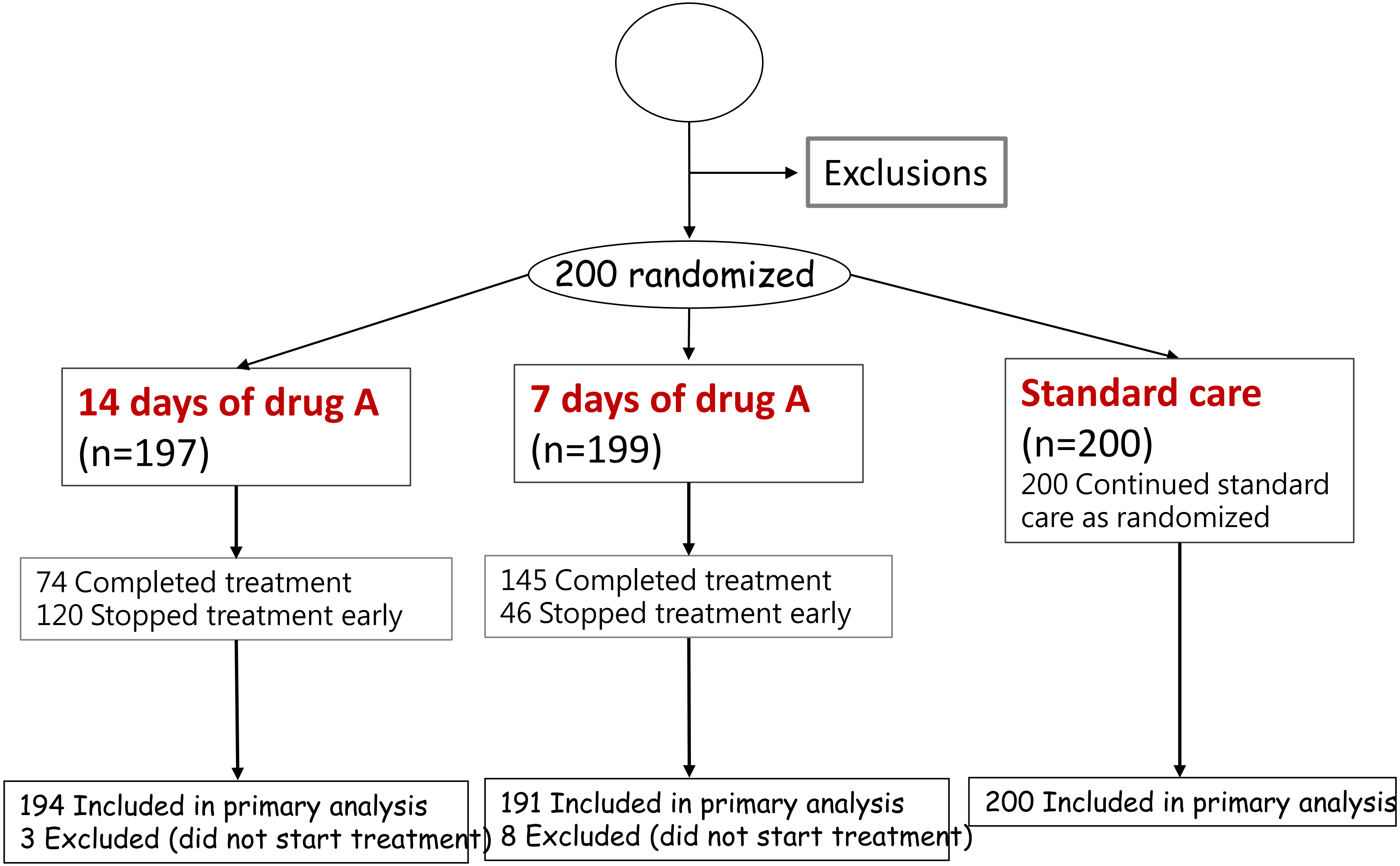

Main outcome: Clinical status on day 15 on a 7-point scale ranging from death (1) to discharge (7). Differences between the two groups were calculated using proportional odds models. An odds ratio greater than 1 indicates difference in clinical status distribution toward category 7 for the drug A group vs the standard care group.

Outcomes	14-Days Drug A (n=194)	7-Days Drug A (n=191)	Standard care (n=200)
Day 15 clinical status n 7-point scale, No.(%)			
1	2(1)	0	4(2)
2	1(1)	0	4(2)
3	0	5 (3)	7 (4)
4	12 (6)	7 (4)	11 (6)
5	44 (23)	38 (20)	46 (23)
6	9 (5)	7 (4)	8 (4)
7	126 (65)	134 (70)	120 (60)
Primary end point: difference in clinical status distribution vs standard care, odds ratio (95% CI)	-	1.65 (1.09-2.48)	1 [Reference]
P value	.18	.02	

Would you agree with the conclusion that 7-days of drug A is effective in managing moderate severity of this condition?

Agree

Doubt

Reject

7 days vs 14 days vs Standard Care

- 30-day Mortality rate
- Pulmonary function
- Hospitalization stay

Clinical status on 7-point scale
1: Death
2: Invasive ventilation with vasopressor
3: Invasive ventilation without vasopressor
4: Noninvasive ventilation
5: Supplemental oxygenation
6: Medication alone
7: No hospitalization

Who would be most appropriate to evaluate the wound one month later?

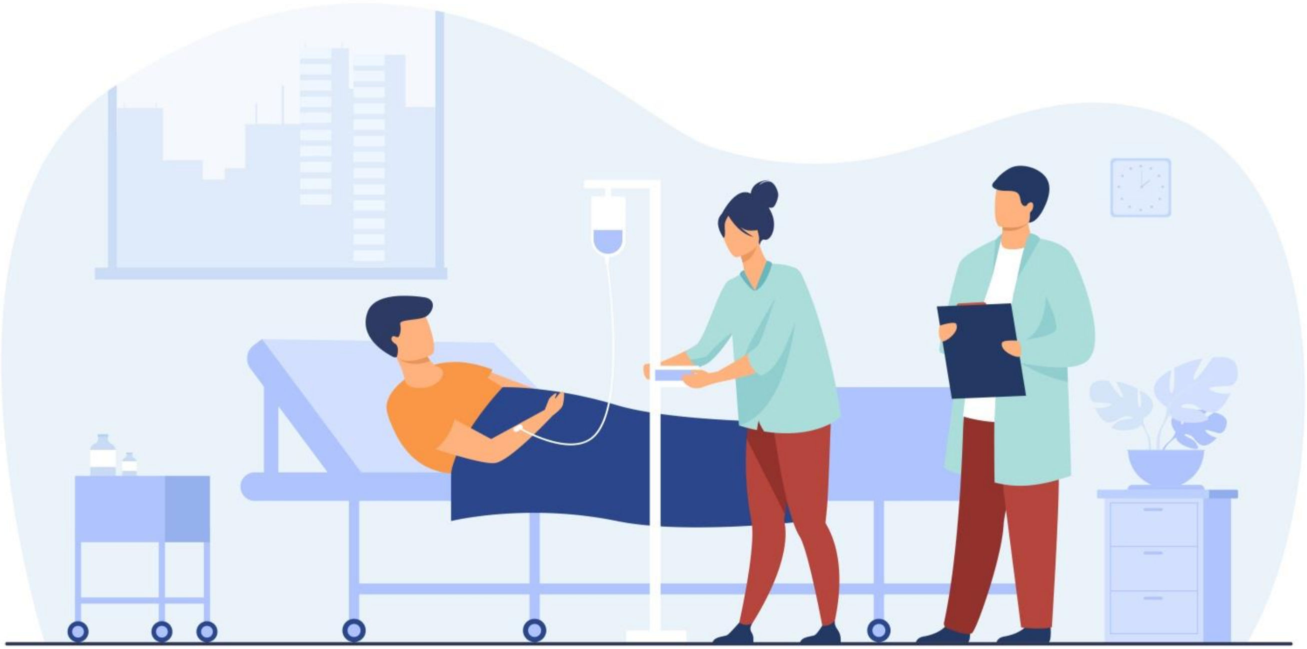

Vote

Attending physician	Resident physician	Ward nurse

Figure S1.4 **Algorithm for suggested judgment of risk of bias in measurement of the outcome.** This is only a suggested decision tree: all default judgements can be overridden by assessors.

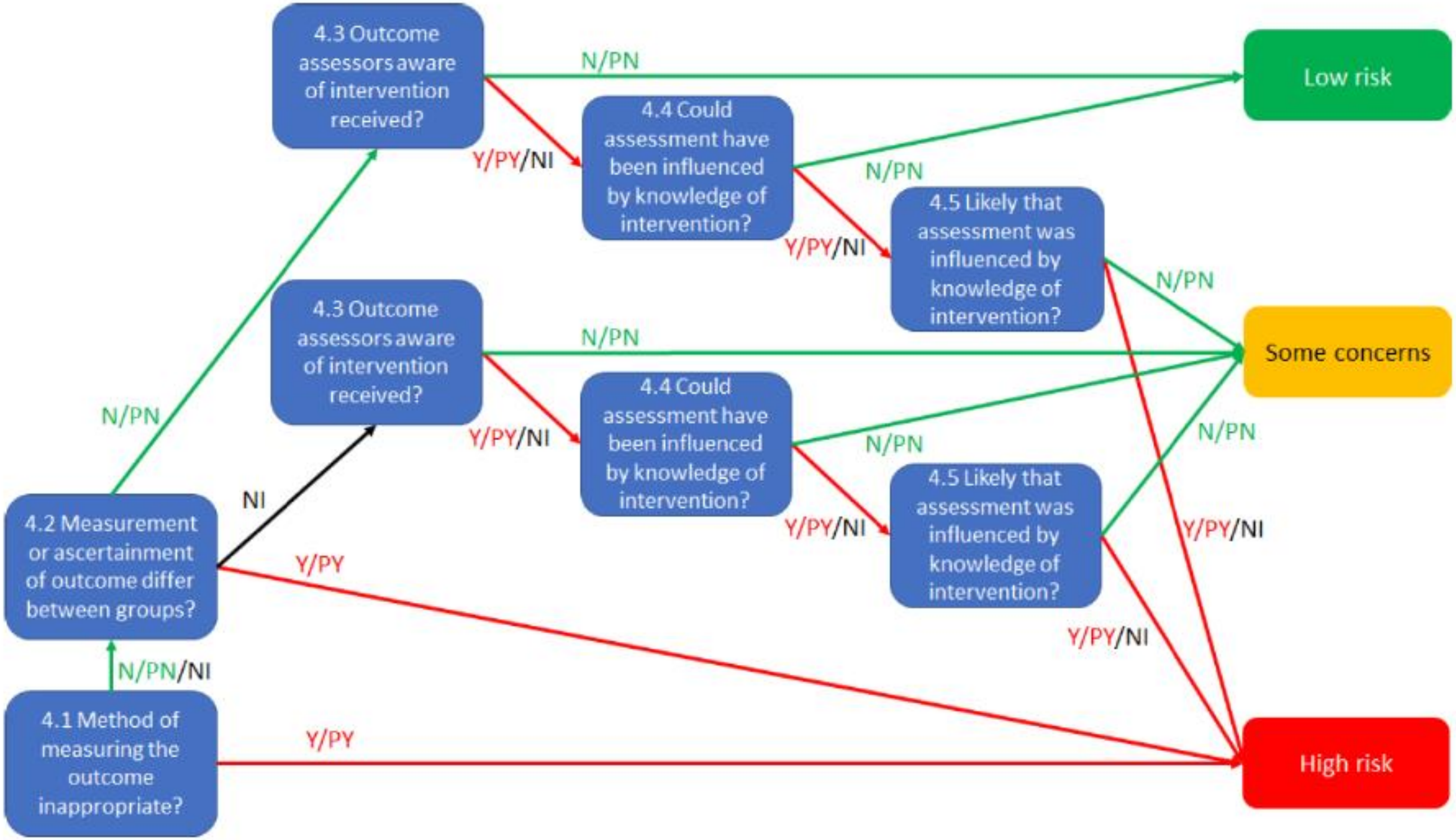

Scenario 11

- You are a professional sports manager, helping the NBA team to buy a shooting guard. You provide the recommendation based on the statistics of the best shooting guards on each team. The most recent stats of shooting guards A and B are presented as follows:

Shooting guard A:

Average points: 30

3-Point field goals made: 18

Shooting guard B:

Average points: 24

3-Point field goals made: 12

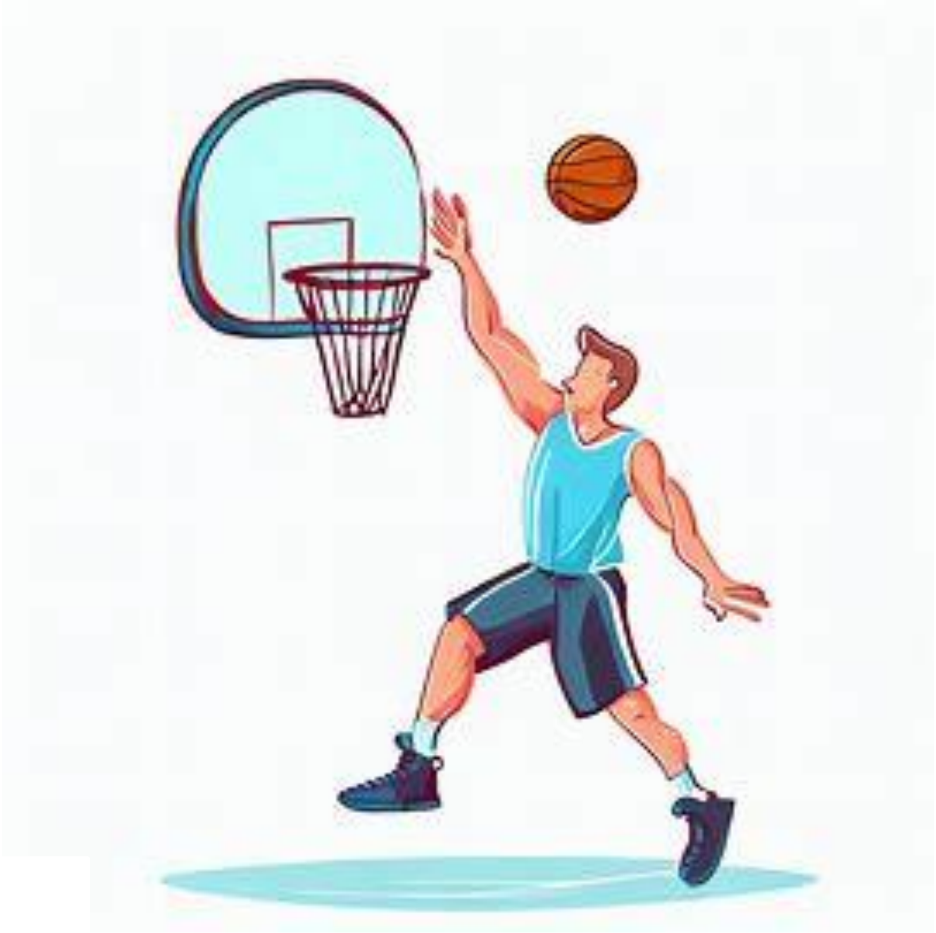

Would you bet on shooting guard A?

Definitely

Doubt

Shooter guard A:

- Average points: 30
- 3-Point field goals made: 18
- Field goal %: 20

Shooter guard B:

- Average points: 24
- 3-Point field goals made: 12
- Field goal %: 50

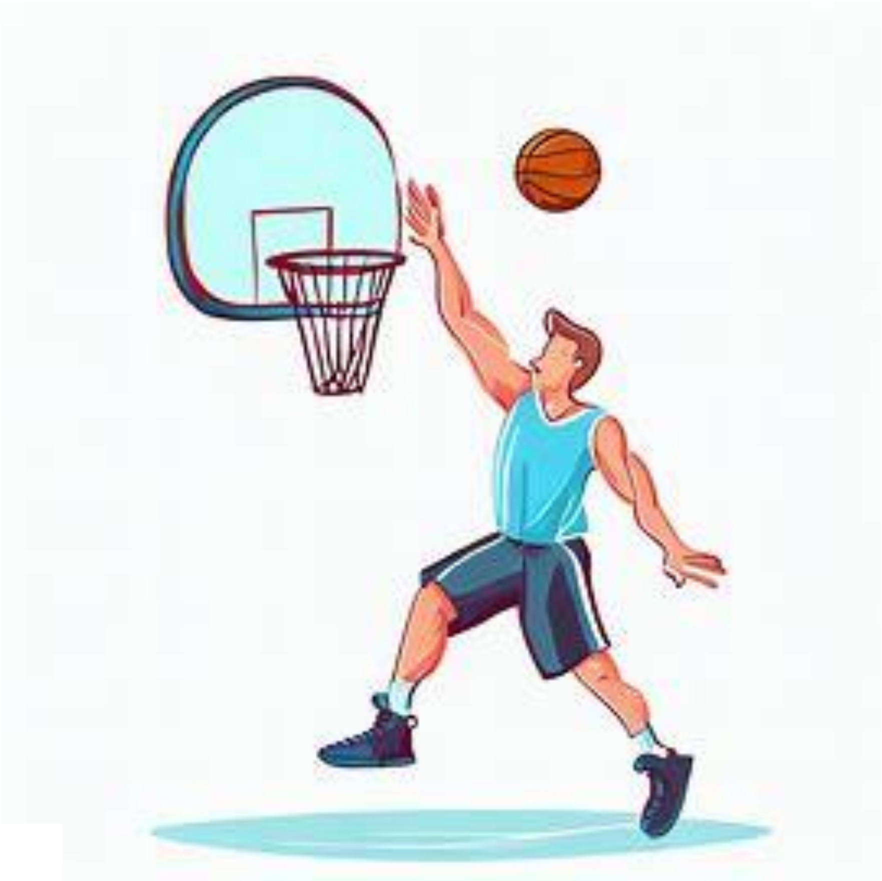

Scenario 12

- You are a researcher investigating the efficacy of a new weight loss medication in obese patients. Upon data collection, you are presented with the amount of weight loss (kg) for each patient, which is as follows:

3,3,4,5,6,7,7,7,7,7,7,7,7,7,8,8,9,10,16,20

What would the most appropriate way to present the data?

Mean ± standard deviation
7.75 ± 3.97

Median (range)
7 (3 to 20)

New weight loss medication

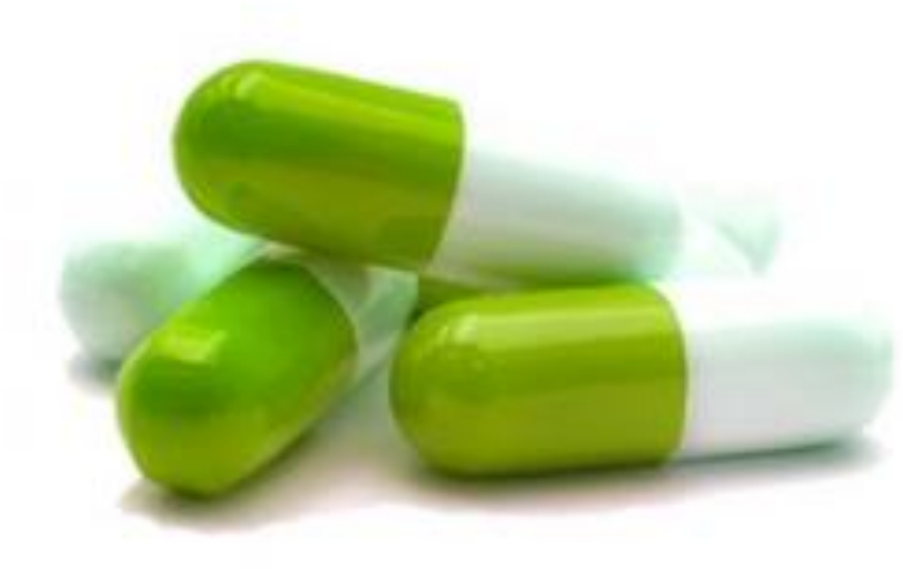

3,3,3,3,3,3,4,4,4,7,7,19,19,20,20,20,20,20,20

Figure S1.5 **Algorithm for suggested judgment of risk of bias in selection of the reported result.** This is only a suggested decision tree: all default judgements can be overridden by assessors.

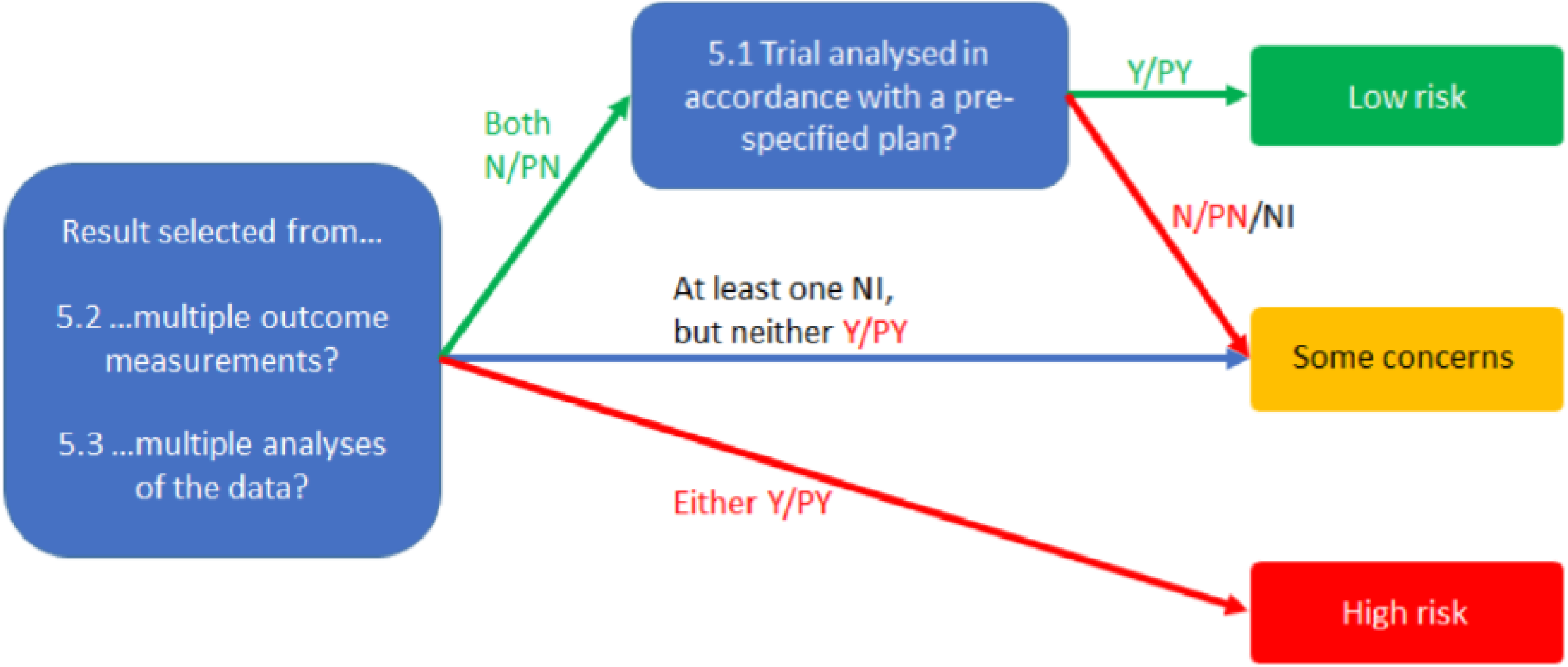

Supplement: Supplementary file 1 — Supplementary Material 1 [file 12909_2023_4738_MOESM1_ESM.pdf]
